# Supplementary material for: Characterization of commercial poultry farms in Mexico: Towards a better understanding of biosecurity practices and antibiotic usage patterns
Source: PLoS One. 2020 Dec 1;15(12):e0242354. doi: 10.1371/journal.pone.0242354 (PMC7707464; doi:10.1371/journal.pone.0242354)
Supplement: S1 QuestionnaireEN — (DOCX) [file pone.0242354.s001.docx]

Epidemiological research questionnaire: risk factors associated with the presence of *Chlamydiaceae* in poultry

Date:

Id. of the farm:

Role of the respondent:

| Farm specifications |  |
| --- | --- |
| 1. Farm location |  |
| 1. What type of poultry houses does the farm have? | □ Open-sided  □ Controlled environment |
| 1. What species of poultry is (are) being bred? | □ Chickens  □ Turkeys  □ Ducks  □ Quails  □ Other: |
| 1. What is the zootechnical function of poultry? | □ Broilers  □ Laying-hens  □ Breeders |
| 1. Is it a specialized breed/strain? | □ Yes  □ No |
| 1. Are other species of poultry bred on the farm? | □ Yes  □ No |
| 1. Which ones? |  |
| Description of facilities |  |
| 1. How many poultry houses are there on the farm? |  |
| 1. What is the number of poultry per house? |  |
| 1. Type of accommodation | □ Ground  □ Litter  □ Cage |
| 1. What is the material used as litter for poultry? |  |
| 1. Are there other domestic animals on the farm? | □ Yes  □ No |
| 1. Which ones? |  |
| Flock specifications |  |
| 1. Sex | □ Male  □ Female |
| 1. Is there sex-separated breeding? | □ 50-50  □ Separate houses  □ No, mixed breeding |
| 1. What is the age of the tested poultry? |  |
| 1. Is there separation of individuals according to age? | □ Yes  □ No |
| Management practices |  |
| 1. What is the duration of the breeding cycle? |  |
| 1. What is the origin of the food? | □ Integrated food plant  □ Commercial food  □ Mix |
| 1. Number of poultry workers on the farm |  |
| 1. Is there a mortality management plan? | □ Yes  □ No (municipal waste) |
| 1. What is the method of disposing of the mortality? | □ Incineration  □ Burial  □ Composting  □ Other |
| Health management and health status |  |
| 1. Do poultry currently show any clinical signs of disease? | □ Yes  □ No |
| 1. Which one(s)? |  |
| 1. Has any antimicrobial treatment been used in this flock? | □ Yes  □ No |
| 1. Which one(s)? |  |
| 1. Please mention how it was used: |  |
| Biosecurity practices |  |
| 1. Is there a perimeter fence? | □ Yes  □ No |
| 1. Is the access to the facilities restricted? | □ Yes  □ No |
| 1. Is there a register at the entrance of the farm for visitors? | □ Yes  □ No |
| 1. Is there a driveway with sanitizing tire bath at the entrance to the farm? | □ Yes  □ No |
| 1. Do people who enter the farm have to take a bath at the entrance? | □ Yes  □ No |
| 1. Do staff who have contact with poultry wear clothing exclusive for the farm? | □ Yes  □ No |
| 1. Do people who have contact with the poultry use protective equipment for handling? | □ Yes  □ No |
| 1. Do people who have contact with birds know what a zoonosis is? | □ Yes  □ No |
| 1. Is there ongoing biosafety training? | □ Yes  □ No |
| 1. Do people who have contact with the poultry have other birds in their homes? | □ Yes  □ No |
| 1. Are there footbaths at the entrance of each poultry house? | □ Yes  □ No |
| 1. Is there a handwashing protocol for staff at the entrance to each poultry house? | □ Yes  □ No |
| 1. Is there any movement/incorporation of poultry before the end of each cycle? | □ Yes  □ No |
| 1. Is there a wildlife/pest control program? | □ Yes  □ No |
| 1. Are there poultry farms within 3 km of the farm? | □ Yes  □ No |
| 1. What type? | □ Commercial  □ Backyard  □ Both |
| Cleaning and disinfection practices |  |
| 1. Is the litter removed at the end of each flock? | □ Yes  □ No |
| 1. Is there a protocol for cleaning the facilities? | □ Yes  □ No |
| 1. Is there a protocol for disinfecting the equipment? | □ Yes  □ No |
| 1. Is there a vacancy period between flocks? | □ Yes  □ No |
| 1. How long does it last? |  |
| Comments/Observations: |  |
