# Supplementary material for: Characterization of commercial poultry farms in Mexico: Towards a better understanding of biosecurity practices and antibiotic usage patterns
Source: PLoS One. 2020 Dec 1;15(12):e0242354. doi: 10.1371/journal.pone.0242354 (PMC7707464; doi:10.1371/journal.pone.0242354)
Supplement: S2 QuestionnaireES — (DOCX) [file pone.0242354.s002.docx]

Cuestionario de investigación epidemiológica: Factores de riesgo asociados a la presencia de *Chlamydiaceae* en avicultura

Fecha:

Id. de la granja:

Función del encuestado:

| Especificaciones de la granja |  |
| --- | --- |
| 1. Ubicación de la granja |  |
| 1. ¿Qué tipo de casetas tiene la granja? | □ Natural  □ Controlado |
| 1. ¿Qué especie(s) de ave es (son) criadas? | □ Pollos/gallinas  □ Pavos  □ Patos  □ Codornices  □ Otra: |
| 1. ¿Cuál es la función zootécnica de las aves? | □ Engorda  □ Postura  □ Reproductoras |
| 1. ¿Es una raza/estirpe especializada? | □ Sí  □ No |
| 1. ¿Se crían otras especies de aves en la granja? | □ Sí  □ No |
| 1. ¿Cuáles? |  |
| Descripción de las instalaciones |  |
| 1. ¿Cuántas casetas hay en la granja?: |  |
| 1. ¿Cuál es el número de aves por caseta? |  |
| 1. Tipo de alojamiento | □ Suelo  □ Cama  □ Jaula |
| 1. ¿Cuál es el material que funciona como cama para las aves? |  |
| 1. ¿Hay otros animales domésticos en la granja? | □ Sí  □ No |
| 1. ¿Cuáles? |  |
| Especificaciones de la parvada |  |
| 1. Sexo | □ Macho  □ Hembra |
| 1. ¿Hay crianza por sexos separados?: | □ 50-50  □ Casetas separadas  □ No, es mixta |
| 1. ¿Cuál es la edad de las aves sujetas a muestreo? |  |
| 1. ¿Hay separación de los individuos de acuerdo a su edad/lote? | □ Sí  □ No |
| Prácticas de manejo |  |
| 1. ¿Cuál es la duración del ciclo? |  |
| 1. ¿Cuál es el origen del alimento? | □ Planta de alimentos integrada  □ Alimento comercial  □ No, es mixta |
| 1. Número de trabajadores en la granja |  |
| 1. ¿Existe un plan de manejo de mortalidad? | □ Sí  □ No (basura municipal) |
| 1. ¿Cuál es la manera en la que se desecha la mortalidad? | □ Incineración  □ Enterramiento  □ Composta  □ Otra |
| Manejo y estado sanitario |  |
| 1. Actualmente, ¿las aves manifiestan algún signo clínico de enfermedad? | □ Sí  □ No |
| 1. ¿Cuál (es)? |  |
| 1. ¿Se ha utilizado algún tratamiento con antimicrobianos en esta parvada? | □ Sí  □ No |
| 1. ¿Cuál (es)? |  |
| 1. Mencione la manera en que fue usado: |  |
| Prácticas de bioseguridad |  |
| 1. ¿Se cuenta con una cerca perimetral? | □ Sí  □ No |
| 1. ¿El acceso es restringido a las instalaciones? | □ Sí  □ No |
| 1. ¿Existe un registro a la entrada de la granja de los visitantes? | □ Sí  □ No |
| 1. ¿Hay un arco sanitario para vehículos a la entrada de la granja? | □ Sí  □ No |
| 1. ¿Las personas que ingresan a la granja deben tomar un baño a la entrada de manera obligatoria? | □ Sí  □ No |
| 1. ¿Las personas que tienen contacto con las aves usan ropa exclusiva de la granja? | □ Sí  □ No |
| 1. ¿Las personas que tienen contacto con las aves usan equipo protector para su manejo? | □ Sí  □ No |
| 1. ¿Las personas que tienen contacto con las aves saben qué es una zoonosis? | □ Sí  □ No |
| 1. ¿Hay entrenamiento continuo en bioseguridad? | □ Sí  □ No |
| 1. ¿Las personas que tienen contacto con las aves tienen otras aves en sus casas? | □ Sí  □ No |
| 1. ¿Hay tapetes sanitarios a la entrada de cada caseta? | □ Sí  □ No |
| 1. ¿Hay un protocolo de lavado de las manos para el personal a la entrada de la caseta? | □ Sí  □ No |
| 1. ¿Hay movimiento/incorporación de aves antes de finalizar cada ciclo? | □ Sí  □ No |
| 1. ¿Existe un programa de control de fauna nociva/plagas? | □ Sí  □ No |
| 1. ¿Hay granjas avícolas a menos de 3 km de distancia de la granja? | □ Sí  □ No |
| 1. ¿De qué tipo? | □ Comerciales  □ Traspatio  □ Ambas |
| Prácticas de limpieza y desinfección |  |
| 1. ¿La cama se retira al finalizar cada parvada? | □ Sí  □ No |
| 1. ¿Existe un protocolo de limpieza de las instalaciones? | □ Sí  □ No |
| 1. ¿Existe un protocolo de desinfección del equipo? | □ Sí  □ No |
| 1. ¿Se hace un vacío sanitario?: | □ Sí  □ No |
| 1. ¿De cuánto tiempo? |  |
| Comentarios/Observaciones: |  |
