# Supplementary material for: Characterization of commercial poultry farms in Mexico: Towards a better understanding of biosecurity practices and antibiotic usage patterns
Source: PLoS One. 2020 Dec 1;15(12):e0242354. doi: 10.1371/journal.pone.0242354 (PMC7707464; doi:10.1371/journal.pone.0242354)
Supplement: Attachment — Submitted filename: Review PONE-D-20-19059.docx [file pone.0242354.s005.docx]

Review PONE-D-20-19059

Characterization of commercial poultry farms in Mexico: towards a better understanding of biosecurity practices and antibiotic usage patterns

Comments:

- PLoS endorsed already many years ago the STROBE guidelines. Please make sure that the reporting of this cross-sectional study complies with the guidelines

<https://journals.plos.org/plosmedicine/article?id=10.1371/journal.pmed.0040297>

- Please provide a justification for the sample size. What coverage of commercial farms do you expect by the 43 farms?
- In line with the sample size, please discuss the robustness of your results for n=43 and 29 variables. I understood that 20 cases for each variable are suggested (<https://doi.org/10.1007/s11135-015-0206-0>). How many veterinarians or farmers refused to participate?
- Did you perform any pre-testing of the questionnaire?
- L 105-107 Please formulate your research hypothesis in more precisely (e.g. similar to l. 25-27).
